# From trucks to tips—examples of peripheral ways by which the meat industry impacts the U.S. workforce and economy

**DOI:** 10.1093/af/vfaa064

**Published:** 2021-05-17

**Authors:** Phillip D Bass

**Affiliations:** Department of Animal, Veterinary, and Food Sciences, University of Idaho, Moscow, ID 83844

**Keywords:** livestock transportation, meat, packing facility, restaurant industry

**Implications**

- Finished meat animal livestock delivery to packers and processors account for an estimated 2.9 million transportation delivery event opportunities providing jobs for those in trucking and associated industries.
- Average production worker earnings in a meat processing facility are over twice the 2020 U.S. federal hourly minimum wage. Not only are those positions earning respectable wages for entry-level positions, but the added worker employment benefits in the meat production and processing industry can enhance the value of the job to the employee.
- A meat entrée on a restaurant menu is typically a higher value-added proposition for the restaurant business and waitstaff than that of a nonmeat dish.
- Meat animal production societal impact is far greater than simply nourishment; it is a system that provides powerful employment and economic opportunities directly and indirectly tied to the system itself.

## Introduction

The meat supply chain impacts far more than just the nourishment of those consuming the product. In the United States, it was estimated that, in 2018, Americans would have had annual access to over 100 kg of red meat and poultry per capita on a retail basis (Jones et al., 2018). Yet, often overlooked is the immense and complex system that is in place to ensure that the wholesome, nutritious, and satisfying food that is meat makes it to the tables in homes and restaurants. From trucking, to processing, to packaging and merchandising, animals, and the resulting meat, pass through many hands and, along the way, a number of industries and lives will be positively affected as a result of meat animal agriculture.

As the system of meat animal agriculture is so vast, it is the intent of this article to compose a small survey of some of those important parts that are integral to keeping the meat industry moving and people consuming meat. This article will provide an economic and personnel employment perspective from that of a meat scientist who, over many years, has had the opportunity to see the inner workings of the meat animal community from gate to plate. The article is constructed to provide broad observations of specific steps in the meat animal agriculture system to help provide context and stimulate discussion on how important of a role meat plays beyond its contribution as food. In this piece, there will be a wide look at how the transportation network for live animals to the packing facilities plays a role in the overall economy of the United States. There will also be an assessment of the labor force needed to keep the meat packing and processing sector operating. Finally, the article will culminate with how meat is an integral part in the restaurant and dining industry using a case study approach, demonstrating how it is ultimately a value-added contributor to those in that service-oriented profession.

## Live Animal Transportation

For anyone who has had the opportunity to observe the large-scale operation of commercial animal harvesting facilities, the immense capacity for transportation that is required to deliver the live animal to the harvest facility and subsequently deliver the finished product to the consumer is quite apparent, even from the first encounter,. Although the evaluation of a large-scale commercial packing plant in North America could lead to a lengthy study in its own right, in order to draw a conclusion as to the immensity of the meat production system, an estimation of the live animal transportation requirements to the packing house can begin to establish a baseline for how impactful the meat production chain is to the transportation industry.

In 2019, the United States slaughtered 26,116,700 young fed beef (steers and heifers), 125,844,300 commercial hogs (barrows and gilts), 2,020,400 sheep and lambs, and 9,224,243,000 broiler chickens under federal inspection (U.S. Department of Agriculture National Agricultural Statistics Service, 2020).

**Figure 1.**
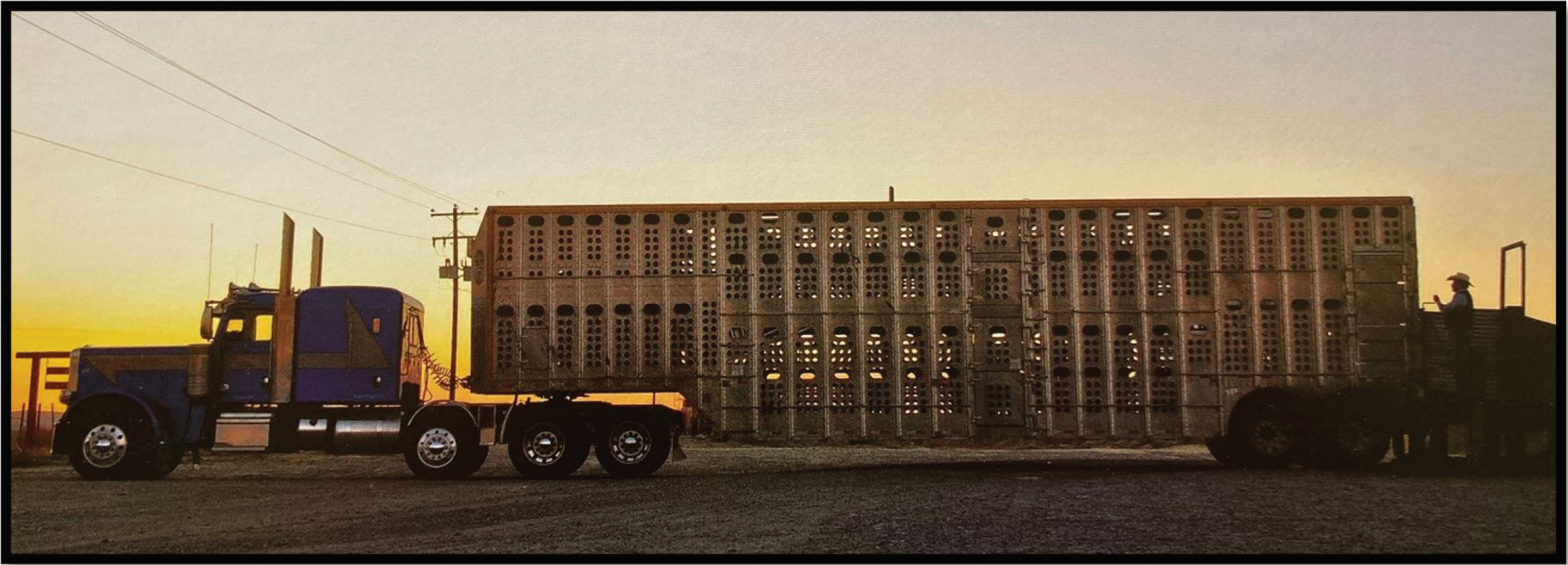
Livestock tractor trailer being loaded with beef cattle (Image credit: Kerner Cattle Company).

According to the U.S. Department of Agriculture Ag Data Commons Animal Transportation Database for Beef Cattle (U.S. Department of Agriculture Ag Data Commons, 2019), a 16.15-m-long livestock tractor trailer with multideck trailer capacity Figure 1 can safely accommodate 38 head of beef cattle with an average live weight of 636 kg. A standard size livestock tractor trailer has an inside trailer compartment dimension of 16.15 by 2.51 m for a floor area of 40.54 m^2^. Many of the commercially operated livestock transporters using the above-mentioned tractor trailer have two layers in the trailer compartment, thereby effectively doubling the floor area capacity available for smaller livestock species, such as hogs and sheep. The Transport Quality Assurance publication recommends 0.45 m^2^/head for hogs with live weights averaging 136 kg each (Transport Quality Assurance, 2019). Therefore, based on those standards, a livestock tractor trailer, as mentioned, would be able to accommodate 180 head of finished hogs per transportation delivery event. Furthermore, using the same size and format of trailer, the recommended density for full-fleece sheep with live weights of 55 kg would be 0.4 m^2^ (Williams, 2000), resulting in approximately 202 finished sheep per delivery event. It is estimated that approximately 6,000 finished broiler chickens can be loaded on a commercial tractor trailer using standard size packing modules stacked 2 high (U.S. Department of Agriculture Animal and Plant Health Inspection Service VS, 2013). Based on the estimated livestock transportation calculations, if one were to use the conservative estimate of a traditional 16.15-m-long livestock tractor trailer with two layers, the annual number of truck delivery events to move the finished beef, hogs, sheep, and poultry to the processing facility would exceed 2.9 million (Table 1).

**Table 1.** Estimated number of total livestock delivery events in 2019 of finished beef, hogs, sheep, and broilers to a packing facility using a 16.15-m-long tractor trailer

Species^*a*^	Number of animals slaughtered under federal inspection in 2019^*d*^	No. of animals per 16.15-m-long livestock tractor trailer	Total estimated number of truck delivery events
Beef^*a*^	26,116,700	38	687,282
Hogs^*b*^	125,844,300	180	699,135
Sheep^*c*^	2,020,400	202	10,002
Broilers	9,224,243,000	6,000	1,537,374
Total			2,933,793

^*a*^Young fed beef steers and heifers, average 636 kg live weight.

^*b*^Barrows and gilts, average 136 kg live weight.

^*c*^Full-fleece sheep, average 55 kg live weight.

^*d*^ U.S. Department of Agriculture National Agricultural Statistics Service (2020).

The aforementioned estimation of trucking delivery events of finished live animals for operation of the U.S. meat processing facilities in 2019 is likely a conservative assessment. The annual total number of transportation events necessary for the harvesting of livestock in the United States is likely larger than the estimated 2.9 million due to a few additional industry dynamic considerations, one in particular would be vehicle size. In the case of processing facilities that harvest older livestock (e.g., cull cows, sows and mutton), the transport of those animals is often done using a pickup truck and smaller trailer, which has much less capacity and would greatly add to the number of transportation events in total for the operation of meat processing facilities in the United States. Regardless of the exact number of trucking delivery events, meat animal agriculture clearly supports a tremendous amount of jobs, directly or indirectly, tied to the transportation industry. Consideration far beyond what could be included in this paper would be related to the economic connection of maintaining the transport vehicles used to deliver the animals to the packing facilities, the fueling network necessary to operate the trucks, the dispatch personnel needed for scheduling the vehicles, and the impact of even the amount of tires sold over time that will require replacement during the lifetime of the transport equipment. Furthermore, consideration could also be made for all of the shipping events and transportation necessities of the finished goods from the meat processing facilities to the final point of purchase. Even though it is only a small snapshot in time, transport of the finished live animal to the processing facility is a significant and necessary economic and employment contributor to the overall food production chain in the United States.

## Packing House Personnel and Material

In 2019, the U.S. Bureau of Labor and Statistics identified that there were 527,460 jobs related to animal slaughter and processing reported (BLS-ASP, 2019). Frontline production workers made up 61.39% of the total number of personnel employed (BLS-ASP, 2019). Support occupations helping to make up the remaining total include but are not limited to transport and material handling, maintenance and repair, office and administrative services, sales, finance specialists, marketing personnel, and technology managers. The majority of those employed at a meat packing or processing facility, however, are skilled technicians who are essential contributors to food production, the economy, and the security of the United States.

A large-scale commercial packing house in North America somewhat resembles a small city at times. Many locations of greater size are far more than a food production facility or place of employment. Meat processing plants are often a place that offers meals, health benefits, and, in most cases, a culture of team spirit that shows just how much can be accomplished when a large group of people work together for a common objective.

Nearly all large-scale packing houses have a cafeteria or dining option on premises that consistently provide hot and cold meals and beverages to employees and at very affordable prices. If a packing house is large enough, it may also provide onsite basic medical services to help maintain worker health and provide for rapid response in the case of a workplace injury incident. Because of the availability of relatively high-paying entry-level positions, the meat production industry has been a destination for many people who are looking for the opportunity of employment, in this case, a trade-based career. With little or no experience, a person working at a packing house can expect to obtain a wage much higher than the 2020 U.S. federal minimum of $7.25/h. (Department of Labor, 2020). In 2019, the mean hourly wage of all meat plant production employees in the United States was $15.20, whereas once an employee advanced through the ranks to a supervisor in production, they could nearly double their hourly wage. The mean wage of meat processing facility production supervisors in 2019 was $27.94/h. (BLS-ASP, 2019). In addition to wages earned, many large-size packing companies offer additional employment benefits, such as health insurance, continuing education classes, discounts on meat products, and valued-worker incentive programs. Considering an hourly plant production worker could begin employment in the United States with no documented education or degree and, in some cases, little to no understanding of the English language, the packing and processing of meat offers a respectable starting career for those interested, willing, capable, and ready to be a part of the food production system workforce.

The packing and processing segment of the meat industry also contributes to other manufacturing industries that supply the necessary tools and consumable products in those facilities. For one example, as reported by the National Beef packing company, a 23.5- × 40- × 60-cm corrugated cardboard box of beef boneless lip-on ribeye subprimals (North American Meat Institute #112A; North American Meat Institute, 2014) would be able to accommodate five subprimals for each case unit (National Beef, 2020). A mid-size beef packing house harvesting 3,500 head of cattle per day would use 1,400 boxes of corrugated cardboard to pack the ribeye product alone. Furthermore, the overwhelming majority of fresh meat in the United States, and other developed nations, is vacuum packaged in a synthetic pouch often made of polypropylene, nylon, polyester film, polyvinylidene chloride, ethyl vinyl acetate, or a composite combination of these materials (U.S. Packaging and Wrapping, 2020; Zhou et al., 2010). Subprimals and cuts, either individually or in combination with others, are vacuum packaged and then subsequently stored and shipped in the corrugated cardboard boxes as mentioned. Packaging materials will make up a large portion of the consumable items in a packing house, which are essential for maintaining food safety, product quality, and overall product yield. Packaging manufacturing companies play an important role in supplying the materials needed to continue to safely and effectively store and distribute meat products to the consuming public; therefore, the meat industry is supporting additional career opportunity in the packaging manufacturing industry beyond those that work directly in a meat processing facility.

Additional resources necessary for equipping the many operational team members in a meat packing and processing establishment include linens (e.g., frocks, aprons, and knit gloves), personal protective equipment (e.g., hard hats, cooler coats, chain mesh gloves and aprons, cut-resistant gloves and sleeves, and hearing and eye protection), and tools of the trade (e.g., knives, scabbards, steels, and saws). Moreover, many of the large packing and processing plants have teams that help to maintain equipment, assist with electronic technology upkeep, manage water treatment and conservation, and have onsite rendering capabilities to utilize the animal and its byproducts to the utmost potential. The economic and employment impact of the overall meat packing and processing community alone is immense in the United States and would be hard to quantify fully considering the complexity of how the industry is formed, as well as how many roles, jobs, and resources it touches.

## Restaurant Dining Industry Impact

The total number of restaurants in the United States in 2018 was surveyed at 660,755 with a total sales estimate of over $800 billion (Miller and Washington, 2020). As an industry, the restaurant business is clearly a large economic contributor and employer; the U.S. Bureau of Labor Statistics reported 10.8 million occupations related to the restaurant industry in 2019 in the United States (BLS-ROEP, 2019). Employment opportunities abound in the restaurant community. Food preparation specialists (chefs and cooks) make up a large part of the worker population in the restaurant industry; however, other positions include servers/waitstaff, dish washers, dining room attendants, food prep workers, and in-house butchers. Moreover, the restaurant business, like many industries, employ a large network of support staff who help to ensure product flow and customer satisfaction; examples of those support positions would include marketing specialists, finance and accounting experts, inventory control managers, market researchers, and customer service personnel, as well as computer and technology technicians. Finally, there is the entire infrastructure that is in place servicing the restaurant community itself that includes distribution specialists, logistics managers, wholesale food and supplies account representatives, and, once again, transportation workers.

Since at least 2010, the National Restaurant Association has ranked, at minimum, one meat-focused category in the top 10 food trends of the year. Eight of those years, the meat category was ranked number 1 and, five of those years, there were two meat categories in the top 10 food trend rankings, indicating that meat cuisine continues to be a major focus for the restaurant community (National Restaurant Association, 2020).

According to Technomic (2018), research into the foodservice protein category found that, in the United States, there were 3.75 billion kg of chicken, 3.65 billion kg of beef and 2.49 billion kg of pork sold at foodservice. Interestingly, in the same study, there was nearly 99% adoption of beef being sold at restaurants (Technomic, 2018). A case study assessment for this article looking at the top 50 independent restaurants in the United States ranked by annual gross income (Miller and Washington, 2020), and the averaged results of their reported online menu pricing for select menu items, is displayed in Table 2.

**Table 2.** Analysis of the average online menu pricing of select item categories from the top 50 independent restaurants in the United States ranked by annual gross revenue in 2019^*a*^

Menu item	Number of menus with those items sold as an entrée (out of 50)	Average menu price of each menu item	$ Gross profit at a 30% menu cost	% Difference from average price of pasta dish	% Difference from average price of salad dish
Beef^*b*^	39	$44.59	$31.22	111	184
Pork	20	$29.20	$20.44	38	86
Lamb	14	$55.60	$38.95	163	254
Chicken	38	$27.66	$19.36	31	76
Burger^*c*^	32	$19.51	$13.66	−8	24
Pasta^*d*^	15	$21.13	$14.79	–	35
Salad^*d*^	41	$15.70	$10.99	−26	–

^*a*^ Miller and Washington, 2020.

^*b*^Specifically selected as filet mignon/tenderloin steak.

^*c*^Ground beef.

^*d*^No meat included.

**Figure 2.**
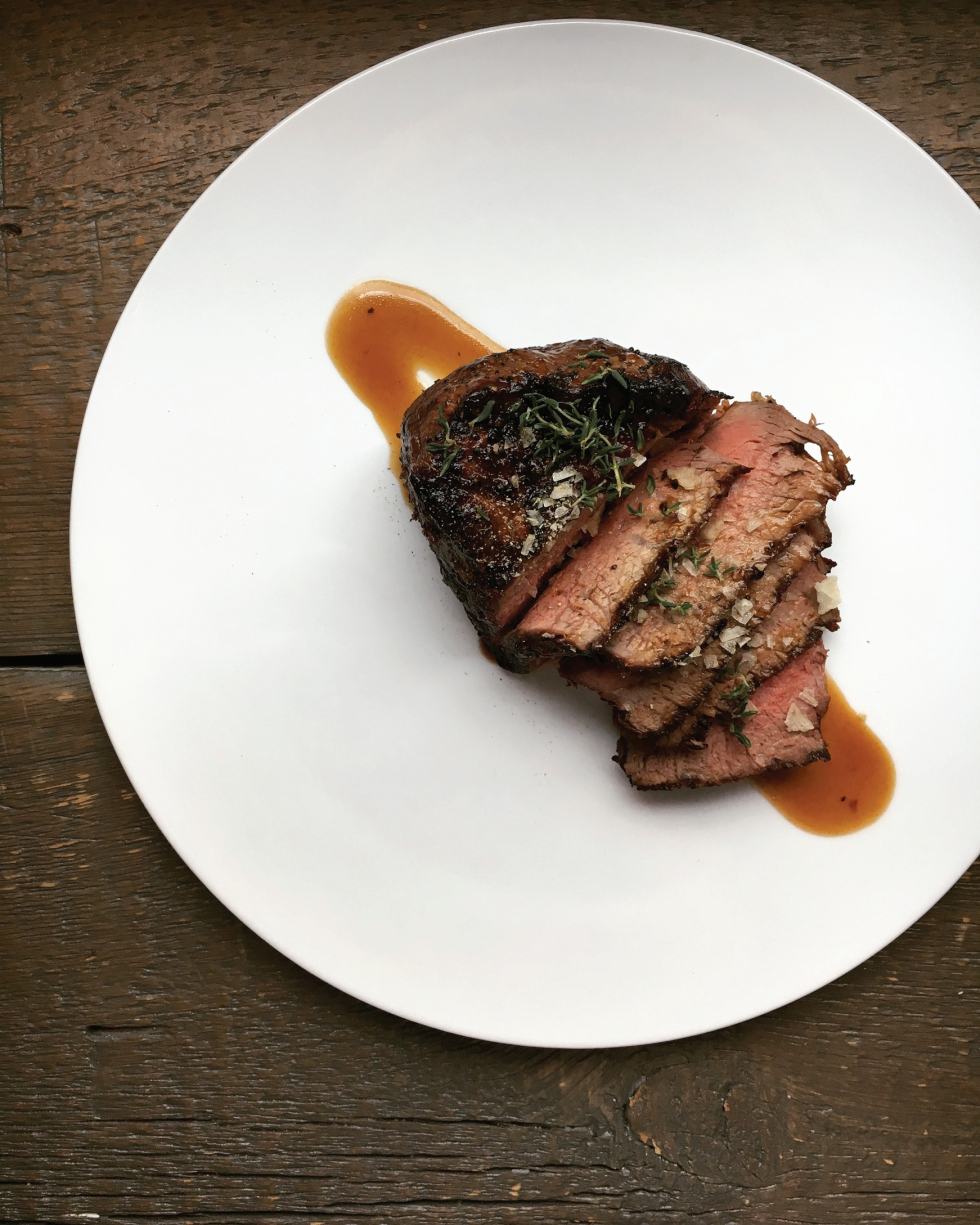
Restaurant plated beef steak entrée (Image credit: Ashley Breneman).

Results of the case study found that all of the top 50 restaurants evaluated had at least more than one meat entrée on their menu (data not shown). All of the meat entrees generated greater gross profit than that of the salad entrée items when the ingredient cost was the commonly assumed 30% of the listed menu price (BC Book Articulation Committee, 2015; Table 2). All of the meat entrees, with the exception of the beef burger, generated greater average gross profit for the restaurant than the nonmeat pasta entrée example (Table 2). Furthermore, if a meat entrée is purchased because the average menu value is consistently higher than that of the nonmeat options, a waitstaff employee could expect a larger tip as tipping in the United States is often based on 15–20% of the total bill (Lynn, 2006). Mean hourly wage for a waitstaff employee in the United States was reported in 2019 as $12.70/h. Tips at a restaurant for a waitstaff employee can be a considerable addition to one’s take-home earnings. A 20% tip for a job well done based on the average beef or lamb entrée menu price reported in Table 2 would be $8.92 or $11.12, respectively. When compared with that of the salad entrée price at a 20% tip equaling $3.14, the incentive to serve the higher valued meat options is over 100% to 200% greater. The ability for meat to generate additional income for the restaurant and waitstaff, as well as provide a tasty meal for the dining customer, demonstrates that its value potential far exceeds that of a vegetable-based option. Meat is a tremendous value-added economic driver in the restaurant industry, which not only generates more profit for the restaurant producing the meal but also allows for additional income potential for employees waiting the tables in those restaurants. Furthermore, meat is considered to have very desirable palatability and aesthetic (Figure 2) characteristics that consumers appreciate, which may help with return business to those restaurants offering a satisfying eating experience. Although the value potential for all restaurants cannot be fully extrapolated from the small exercise demonstrated in Table 2, it could still be assumed that, relatively speaking, a meat entrée will continue to be a greater take-home finance generator for a restaurant business than a nonmeat meal.

## Summary

**Figure 3.**
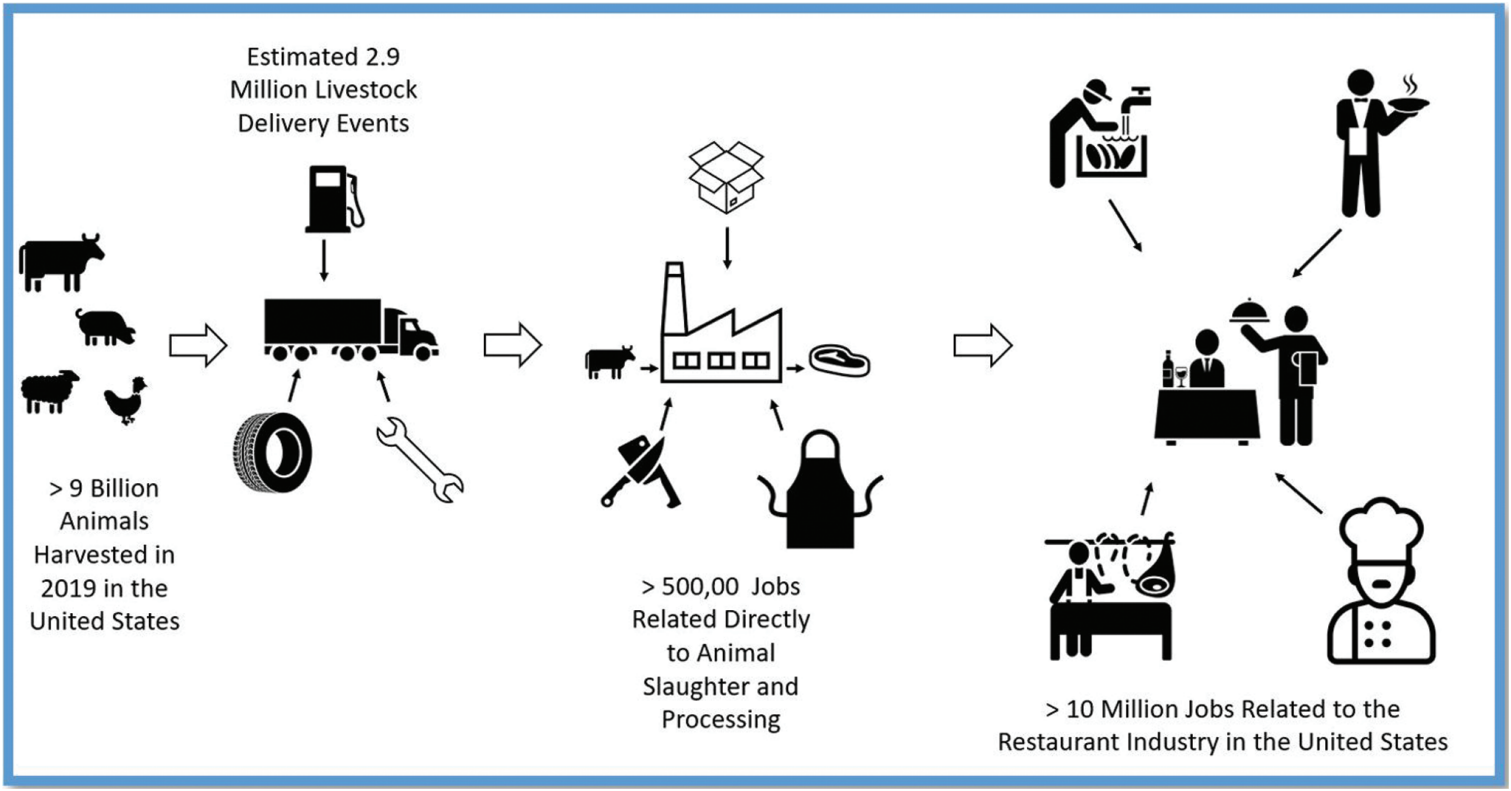
Diagram of industry connections to the meat animal livestock system.

The meat animal industry is an immense network of workers and resources (Figure 3). From the point of delivery of a live finished animal to the packing facility to the point of consumption at the home or restaurant, the meat industry as a whole impacts millions of jobs. If one were to consider the greater immensity of the meat animal sector and backtrack to the farmers and ranchers raising the animals for food production, it would increase that number even further. The meat animal itself is an incredible value-adding entity in that, in most cases, it converts either low-value or, in the case of ruminants, indigestible, vegetable material into nutritious and palatable protein products that continue to be in great demand in both the United States and beyond.

There continues to be tremendous pressures by a number of groups that tout the virtues of limited meat consumption or even suggesting the curtailing and harvesting of all animals for meat. It is important to realize the immense human benefit of meat animal production and how many jobs, people and processes it is tied to, not to mention the value-added economic impact it supplies. Furthermore, meat provides a satisfactory addition to a meal that enhances the human experience for those who are able to consume it for whatever reason. Meat animal agriculture and meat production provide far more than nutrition for a growing U.S. and global population. Although this article focused on the U.S. meat animal model, many of the major players in the group of industrialized nations also have processes in place similar to that of the United States and, as a result, meat will continue to be a hub in a very large network.

*Conflict of interest statement.* None declared.

## About the Authors

**Phil Bass** earned his bachelor’s and master’s degree in animal science from California Polytechnic State University and his PhD in meat science from Colorado State University. Phil served as the Senior Meat Scientist for the Certified Angus Beef brand for several years where he led educational programs and analyzed data for the betterment of the brand and beef community. Phil transitioned to a faculty position in the Animal, Veterinary and Food Sciences Department with the University of Idaho in 2017 where he teaches and focuses his research primarily on ways of improving meat animal carcass value and palatability through practical and applied means.
